# Alveolar Regeneration in COVID-19 Patients: A Network Perspective

**DOI:** 10.3390/ijms222011279

**Published:** 2021-10-19

**Authors:** Shishir K. Gupta, Mugdha Srivastava, Rashmi Minocha, Aman Akash, Seema Dangwal, Thomas Dandekar

**Affiliations:** 1 Functional Genomics & Systems Biology Group, Department of Bioinformatics, Biocenter, Am Hubland, University of Wuerzburg, D-97074 Wuerzburg, Germany; mugdha.srivastava@uni-wuerzburg.de (M.S.); aman.akash@stud-mail.uni-wuerzburg.de (A.A.); 2 Core Unit Systems Medicine, University of Wuerzburg, D-97080 Wuerzburg, Germany; 3 Department of Biochemistry, All India Institute of Medical Sciences, Ansari Nagar, New Delhi 110029, India; rashmi.phd.2012@gmail.com; 4 Stanford Cardiovascular Institute, Department of Medicine Stanford University School of Medicine, Palo Alto, CA 94305-5101, USA; sdangwal@stanford.edu; 5 BioComputing Unit, European Molecular Biology Laboratory (EMBL), Meyerhofstraße 1, 69117 Heidelberg, Germany

**Keywords:** COVID-19, SARS-CoV-2, alveolar regeneration, alveolar fibrosis, signaling pathway, network biology

## Abstract

A viral infection involves entry and replication of viral nucleic acid in a host organism, subsequently leading to biochemical and structural alterations in the host cell. In the case of SARS-CoV-2 viral infection, over-activation of the host immune system may lead to lung damage. Albeit the regeneration and fibrotic repair processes being the two protective host responses, prolonged injury may lead to excessive fibrosis, a pathological state that can result in lung collapse. In this review, we discuss regeneration and fibrosis processes in response to SARS-CoV-2 and provide our viewpoint on the triggering of alveolar regeneration in coronavirus disease 2019 (COVID-19) patients.

## 1. Introduction

The novel coronavirus disease 19 (COVID-19), caused by the highly pathogenic SARS-CoV-2 (Severe acute respiratory syndrome coronavirus 2) virus, has resulted in more than 234 million infections and about 4.8 million global deaths up until October 2021, according to the World Health Organization (WHO) [1]. The SARS-CoV-2, which stands for severe acute respiratory syndrome coronavirus-2, along with the previously existing SARS-CoV and Middle East respiratory syndrome coronavirus (MERS-CoV) comprise a group of human coronaviruses that have affected human population adversely in the last two decades. All of them invade by infecting the cells in the upper respiratory tract along with the bronchial epithelial cells as well as pneumocytes, the result of which is a severe lung infection [2]. Every viral infection involves entry and replication of viral nucleic acid. Specifically, SARS-CoV-2 is an RNA virus.

The outcome of critical SARS-CoV-2 infection during the on-going pandemic has been found to be highly destructive and life-threatening for the host organism. It has been established that SARS-CoV-2 primarily affects the respiratory tract and in severe circumstances this virus can induce pneumonia and acute respiratory distress syndrome (ARDS) as well as several extrapulmonary manifestations. ARDS is a severe form of lung injury characterized by noncardiogenic pulmonary edema, bilateral pulmonary infiltrates and profound hypoxemia leading to respiratory failure. In the case of an advanced stage of lung injury or delayed diagnosis, even antibiotic treatment can become insufficient to avoid development of acute lung injury [3]. The population-based incidence of ARDS has been estimated to range from 10 to 86 instances per 100,000, but it is also believed that the condition is underdiagnosed especially in low-income countries [4]. Additionally, ARDS is a life-threatening illness with a high mortality rate [5]; there have been very modest improvements in recent decades, and increased mortality in the elderly [6]. Patients with ARDS frequently show diffuse alveolar damage (DAD) [7]. The acute phase of ARDS results from DAD and endothelial injury, while late phase is associated with proliferation of alveolar epithelial type2 (AT2) cells and fibroblasts, followed by chronic inflammation and extensive pulmonary fibrosis of the alveoli leading to a loss of normal lung architecture [4]. Of note, with the promotion of fibrosis, reestablishment of normal lung architecture is extremely challenging in human.

ARDS has also been established as one of the major hallmarks of severe COVID-19 infections. In fact, around 40% of COVID-19 patients developing pneumonia also developed ARDS and around 70% of deaths in COVID-19 infected critically ill patients occurred as a result of ARDS [7]. However, the disease when it occurs as part of COVID-19 behaves differently and there is insufficient data to explain explicitly how pathophysiology of COVID-19 ARDS is different from that of the typical ARDS [8]. A clinical study compared the histopathological and molecular features of lungs obtained from autopsies of patients who died from a COVID-19 infection to those who died from ARDS after an influenza A infection. Although similar morphological patterns were observed in both types of lungs, unique vascular features such as severe endothelial cell injury, extensive vascular thrombosis and a higher degree of vascular angiogenesis were particularly noticed in the COVID-19 infected lungs [9]. Thus, there is a pressing need for finding an effective treatment for COVID-19 ARDS, which is also a current challenge for doctors and researchers. 

Even though the on-going SARS-CoV-2 pandemic has unprecedentedly and severely impacted our health-care system and economic growth, a conclusive cure for it still does not exist. Though the majority of COVID-19 cases are mild, around 5–8% of patients develop life-threatening critical illnesses. The vaccination drives for COVID-19 are in progress in most countries, and seemingly, it may require a considerable amount of time before the vaccine reaches all over the globe. Additionally, growing numbers of incidents have been reported worldwide, where patients who survived the illness of COVID-19 and tested negative clinically, still continue to struggle through the symptoms of the disease and post virus-clearance complications, including significant lung damage and cardiac arrhythmias [10,11]. Several autopsy studies of patients who died of COVID-19 have reported extensive alveolar damage to the lungs, while long-term lung impairment has been reported in the survivors of COVID-19 disease [11]. Thus, there is a critical need for a deepened understanding of the biomolecular mechanisms that govern these virus–host interactions so that effective preventive, as well as treatment strategies, can be developed for post-COVID-19 lung infections. Notably, in order to restore normal functioning of lungs post-COVID-19 infection, a highly regulated inflammatory response is usually triggered in the host organism. Alveolar regeneration and fibrotic repair are two main processes by which a host organism can heal the damaged tissue after injury. Both processes are tightly regulated and involve crosstalk between different cell types. Hence, understanding of key proteins and cellular pathways underlying regenerative cells can be helpful to identify the drug targets for pathological intervention. In this review, we summarize our current understanding of the regeneration and fibrotic repair processes, especially in the context to the SARS-CoV-2 infection. Furthermore, we emphasize the network biology potential and demonstrate its applicability in order to understand the mechanisms of regeneration switching. 

## 2. Alveolar Regeneration

The lung, despite its general quiescent state, has a remarkable regenerative capacity, in which resident progenitor cell populations proliferate and differentiate into a variety of cell types in response to injury [12]. Recent evidence suggests that the lung contains several spatially and temporally restricted progenitor cells capable of producing all types of pulmonary cells [12]. Among these, alveolar type I cells (AT1) and alveolar type II cells (AT2) constitute two major cell populations found in the alveolar epithelium [13]. During lung injury, AT2 cells behave as adult tissue stem cells and play a critical role in tissue regeneration by differentiating into AT1 cells [14,15,16,17,18]. Notably, despite being the most abundant cells in the alveolar space, AT2 cells only cover ~5% of the total surface due to their unique cuboidal morphology [19]. The remaining ~95% of the surface is covered by large squamous attenuated AT1 cells. Bronchial and alveolar epithelial cells are key targets of viral pathogens. SARS-CoV-2 can infect both AT1 and AT2 cells ex vivo [20]. Long-term infections can result in uncontrolled inflammation and lung damage. Together with ciliated airway cells, AT2 cells are primary targets for SARS-CoV-2 infection [21,22]. A detailed examination of lungs from patients who died from SARS-CoV-2 infection demonstrated that AT2 cells were the most extensively proliferating cell population in severely damaged lungs. This suggests that AT2 cells might be implicated in the alveolar regeneration following SARS-CoV-2 infection [23]. In other words, the alveolar regeneration capability may be directly modulated by the SARS-CoV-2 infection. However, limited information exists so far on the molecular mechanisms involved in repairing and rebuilding an operational respiratory system after lung injury due to SARS-CoV-2. 

To date, rodent models have been the most effective for providing direct experimental evidence for in vivo alveolar regeneration. However, despite the conservancy in lung development among mammals, the structure, cellular composition, molecular mechanisms, and reactions of mouse and human lungs are vastly different [24,25]. In rodents, complete alveolar regeneration, and restoration of lung functions after injury takes only a few weeks [26,27]; whereas in humans, it requires a very long time scale. There is a study which showed that a 33-year-old woman who had undergone pneumonectomy had complete alveolar regeneration over a period of 15-years [28]. Intriguingly, several recent studies have also confirmed the enrichment and implication of various types of progenitor cells in the regeneration of damaged respiratory epithelium following a SARS-CoV-2 infection. For instance, Chen et al. (2020) showed evidence of alveolar regeneration in 54-year-old and 58-year-old COVID-19 patients [26]. In these patients, AT2 cells could differentiate into AT1 like cells, although the regeneration process began on the 38th day after the commencement of the first symptoms [26]. In another study, tracheas and lungs from five COVID-19 deceased patients were examined. Interestingly, an extensive population of proliferating Krt5^+^ basal cells was found to be enriched in the trachea and larger airways. In addition, a population of extensively proliferating AT2 cells was observed in the intrapulmonary airways and alveoli of these patients. This suggests that distinct populations of proliferating progenitor cells become enriched at the area of lung damage in order to regenerate the damaged trachea and alveoli following SARS-CoV-2 infection [23]. Zhao et al. (2020) also identified a possible mechanism of lung repair following severe SARS-CoV-2 infection [29]. Their single-cell RNA-sequencing analysis confirmed a significant increase of lung progenitor Tm4sf1+ and Krt5^+^ cells in critical COVID-19 patients, both of which could act together synergistically to restore epithelial barriers and regenerate alveolar cells [29]. One other investigation assessed the magnitude of lung regeneration after recovery from SARS-CoV-2 infection [30]. It was found, through an analysis of serum cellular markers in COVID-19 infected patients, that AT1 cellular damage was no longer discernible during the recovery period after virus clearance. Conversely, AT2 cells, as well as lung structures, were still found to be damaged two weeks after clearance of SARS-CoV-2 [30]. 

## 3. Alveolar Fibrosis

The regenerative process is often capable of restoring the function and structure of the lung after damage. In the case of prolonged and pervasive damage, the lung is healed by an accumulation of fibers at the site of injury. Although this fibrotic repair mechanism produces scar tissue, it gradually results in epithelial and endothelial damage causing a significant loss of lung function and an increase in morbidity [31]. Pulmonary fibrosis is a common result of most chronic inflammatory lung disorders, and it can have an impact on lung function, ultimately leading to its failure and death [32]. Fibrosis is scarring of the affected tissue due to the accumulation of extracellular matrix (ECM) components such as collagen and fibronectin but in abnormal excessive amounts. The transformation of normal repair to fibrosis also depends on the severity and duration of the damage as long-lasting damages tend to develop fibrosis as compared to small-scale injuries [33,34].

It has been established that interactions between different types of cells is very crucial for the onset of fibrosis. In pulmonary fibrosis, mesenchymal cells and fibroblasts are recognized as key cell types which partake in heavy ECM deposition, leading to fibrosis and decreased lung function [35]. These cells undergo a lot of changes after an event of tissue damage. Activation of glycolysis in fibroblasts after lung damage initiates a cascade of enzymatic activations which elevate cell proliferation, collagen synthesis and production of secondary metabolites thereby promoting fibrosis. Increased glutaminolysis and fatty acid oxidation play an important role in fibroblast activation. Inflammatory monocytes and tissue-resident macrophages are also critical regulators of tissue fibrosis, helping to initiate, maintain, and resolve tissue damage. Macrophages are also involved in the recruitment of fibroblasts which leads to fibrosis. In addition, the severity of fibrosis depends on factors such as age, genetics, and environmental factors [36]. Recent studies have revealed the significance of epithelial cells in fibrogenesis. Epithelial cells under special circumstances undergo transformation and gain fibroblast-like properties by a process known as epithelial-mesenchymal transition. These cells can migrate to the site of inflammation and contribute to fibrogenesis. Thus, all these cell types interact and contribute to tissue repair but any disparity in their normal activity or function can lead to fibrosis [37]. In other words, fibrosis is an outcome of any shortcoming in the cellular crosstalk during the tissue repair process. Notably, chemokines play a central role in the onset of fibrosis especially the transforming growth factor (TGF-β1), vascular endothelial growth factor (VEGF), platelet-derived growth factor (PDGF), and fibroblast growth factor (FGF). TGF-β1 especially plays a central role in pulmonary fibrosis by promoting the production of ECM as well as by regulating its own expression. It is further regulated by different cell types which serve dominant roles in fibrogenesis and include fibroblasts, macrophages, and epithelial cells [38].

Viral infections play a key role in fibrogenesis and increase the risk of fibrosis [39]. These infections cause severe inflammation and can pair with genetic factors, as well as old age, eventually leading to fibrosis. Pulmonary fibrosis is also central to the SARS-CoV-2 infection, as indicated by the available radiographic, autopsy or other emerging clinical evidence [40,41,42]. In COVID-19 patients, alveolar damage and changes in the fibroblast niche are two prime causes of pulmonary fibrosis [43]. The damage to the alveolar epithelium can stimulate the injured cells to release molecules such as damage-associated molecular patterns (DAMPs) that are recognized by macrophages which further trigger downstream inflammatory responses including activation of toll-like receptors (TLRs) and inflammasomes as well as the release of cytokines such as IL-1 and TNF [41,44,45]. The damage further leads to the stimulation of endothelial cells and endothelial leukocyte adhesion molecules (ELAMs) that recruit leukocytes to the site of damage. Following alveolar damage, fibroblast growth factor (FGF) along with TGF-β1 and chemokines are also stimulated, leading to the recruitment of fibroblasts to the site of injury. These fibroblasts then proliferate and differentiate into myofibroblasts, further activating the inflammatory responses, including epithelial or endothelial to mesenchymal transition. The accumulation of fibroblasts and myofibroblasts can thus lead to over-active secretion of extracellular matrix components. Altogether, dysregulation in any of these response elements as well as aberrant accumulation of various chemokines, pro-fibrotic growth factors, and myofibroblasts can disrupt a well-organized healing response eventually leading to pulmonary fibrosis. There are an increasing number of reports of pulmonary fibrosis in COVID-19 patients as confirmed by radiological scans. For instance, in two different studies conducted by Zhou et al. and Pan et al., chest CT scans showed fibrotic changes especially during the advanced phase of the disease [46,47]. Furthermore, autopsy reports of patients who died due to COVID-ARDS or post-COVID pneumonia confirmed extensive alveolar damage as well as lung fibrosis [48,49,50]. Yu et al. (2020) compared the CT findings and clinical features of COVID-19 discharged patients who did or didn’t develop pulmonary fibrosis and reported that fibrosis is the more likely outcome in patients who are old age and have serious clinical conditions including an increase in inflammatory indicators CRP and IL-6 [51]. Thus, SARS-CoV-2 induced lung fibrosis may altogether modify lung biomechanics resulting in irreversible tissue damage [52]. However, a direct relationship between viral infection and pulmonary fibrosis and molecular mechanisms involved in this process are still not apparent [53]. At the RNA level, the downregulation of miR203 might be a key factor involved during pulmonary fibrosis [54]. Another key factor is the transcriptional factor SNAl1 which plays a direct role in epithelial to mesenchymal transition (EMT) and fibroblast activation. SNAl1 is regulated by TGFβ which, as discussed above, plays a key role in fibrosis and causes EMT, which is crucial to fibrosis [55]. Genome wide studies are further required in order to identify specific genes associated with post-COVID-19 lung fibrosis.

## 4. Fibrotic Repair Is Dominant over Regeneration

The level of inflammation in the lungs following tissue injury is a crucial factor to determine whether regeneration or fibrotic repair will take place [56]. Both repair processes are often sequential and interrelated [57]. In the case of limited damage, regeneration usually occurs first to restore the integrity and function of the tissue. If this process fails because of severe damage, fibrotic repair initiates. Again, if the fibrotic repair fails or if there is excessive fibrotic repair in response to high levels of injury or inflammation, it may lead to chronic lung disease or collapse. This suggests that regeneration is a better healing process while repair is only good when it is moderate. Unfortunately, in the case of virus induced severe infection, fibrotic repair is the dominant process over regeneration [31,53]. 

Due to lack of post-injury lung samples, suitable animal models, and regeneration-specific molecular markers, alveolar regeneration in humans is still not well characterized. In this pursuit, single-cell RNA sequencing (scRNA-seq) technology has revealed the cellular architecture of the lung and the intermediate state of AT2 to AT1 transition known as Krt8+ cells [17]. AT2 and AT1 cells are classes of distal airway stem cells and Krt8+ cells are the transitional stem cells between AT2 and AT1 as mentioned before. Krt8+ cells appear in both small and big injury and normally peak at 10 days after the injury. They also have the ability to transition back to AT2 cells. These cells are distinguished by their high expression of keratin 8 and distinct gene sets having some similarities with AT1 cells. In the case of injury, these progenitor cells are expressed in the damaged tissue in high numbers. Apart from AT2 cells, activated club cells also play a role in the differentiation to Krt8+ cells by transcriptional regulation in these two stem cells but the detailed mechanism behind this process is still not clear [14,17,58]. After injury, all these cell types, including AT2 cells, Krt8+ cells, and activated club cells, undergo proliferation leading to fibrosis. Krt8+ cells were also found to have an affinity for macrophages, fibroblasts, and endothelial cells. In addition, they also secrete many factors involved in fibrogenesis. Accumulation of Krt8+ cells and not transitioning to AT1 cells can actively lead to fibrosis confirming that Krt8+ cells are profibrogenic. Krt8+ cells were found to be interacting with the cellular niche to coordinate the regeneration process. However, Krt8+ cells do not express similar ligands and receptors to its endpoint AT1 cells and thus are considered a different cell lineage. However, they play an essential role in processes such as inflammation, angiogenesis, and fibrogenesis [15,16,17].

Given the increasing number of incidences of post-COVID lung fibrosis, it is paramount to understand the underlying molecular mechanisms and develop effective targeted strategies for its treatment. A potential approach in this regard is to design therapeutic approaches that can promote regeneration over repair mechanisms. For instance, use of corticosteroids such as dexamethasone indirectly affects this by delaying the repair rate and reducing inflammation [59]. The successful use of dexamethasone in reducing mortality in severe COVID patients has been demonstrated in many trials already [53,60]. Another approach would be to design anti-fibrotic therapeutic strategies in order to slow down the rate of fibrotic progression [61,62]. The two anti-fibrotic drugs already available commercially and currently being tested in COVID-19 patients are pirfenidone and nintedanib [52]. It has also been suggested to use anti-inflammatory drugs such as steroids in combination with the anti-fibrotic drugs as a better treatment strategy [63]. A study performed in mice demonstrated that the blocking of platelet-derived growth factor receptor β (PDGFR-β), where the growth factor PDGF has been shown to be involved in the pathogenesis of fibrosis, reduces the effects of pulmonary fibrosis [64]. It would be further intriguing to perform similar studies using animal models in order to investigate post-COVID lung fibrosis. Moreover, inflammatory cytokines such as IL6, which play an important role in the development of lung fibrosis, might also act as potential therapeutic targets for its treatment. We hypothesize that understanding the cellular network of AT2 cells could be an important step to elucidate critical factors involved in state transition.

## 5. Role of miRNAs as Markers and Therapeutic Targets for COVID-19

miRNAs (micro-RNAs) are short (~22 nucleotides) non-coding RNAs which perform regulatory functions in the cell by targeting mRNA. Specific miRNAs of a host organism are differentially expressed when infected to promote viral RNA destruction and to control the immune response. From the pathogen side, viral-derived miRNAs play a role in immune evasion and their regulation of the viral cycle, and may also contribute to tumorigenesis [65,66]. In the COVID-19 infection, miRNAs may act as markers for the identification of the SARS-CoV-2 infection. In infected patients, several human miRNAs have been reported to be upregulated up to 50 times when compared to healthy donors. Based on these differences in expression levels, these miRNAs can be potentially employed to identify SARS-CoV-2 infections with an accuracy of more than 99 percent. It has also been observed that miRNAs regulating inflammation and cytokine expression are among the ones that are differentially expressed in the presence of SARS-CoV-2 infection [67]. Regarding the viral pathogen, annotation of SARS-CoV-2 derived miRNAs has revealed the effect of viral miRNAs on host transcriptomics. SARS-CoV-2 miRNAs may affect pathways involved in insulin signaling, heart development, and brain functions. Cellular maintenance and regulation may also be negatively affected by SARS-CoV-2 miRNAs during hypoxia, a condition leading to death in COVID-19 patients [68,69]. Apart from the role of miRNAs in host–pathogen interaction, miRNAs are also being investigated as therapeutic targets. The major cause of mortality in COVID-19 patients is lung dysfunction due to pneumonia leading to alveolar damage resulting in severe hypoxemia [70,71,72]. Lung alveolar regeneration to repair the damaged tissue and restoration of normal tissue function could be achieved by transplantation of progenitor or stem cells [73,74] and exosome-mediated delivery of therapeutic agents, including miRNAs [75,76]. Not only as a biomarker of COVID-19 [77,78] but also as therapeutic agents, miRNAs have proven to play a crucial role in lung damage and repair [76,79,80,81]. miRNAs can either be regulated locally in the lungs [82] or transported to the damaged site by extracellular vehicles (EVs) secreted by stem cells to induce tissue regeneration by decreasing inflammation and apoptosis, stimulating surfactant production, regulating gene expression of junction proteins to repair microvascular permeability, and reducing fibrosis [75,76]. As a striking example, the role of miR-30b-3p, miR-27a-3p, miR-145, miR-302, and miR-486 have been reported to be essential in alveolar repair. Yi et al. (2019) demonstrated that elevation in EVs associated miR-30b-3p concentrations increased alveolar epithelial cell proliferation and reduced apoptosis via a decreased pro-inflammatory mediator, SAA3 [79]. Similarly, Li et al. (2018) suggested the role of miR-486 in reducing apoptosis in human lung alveolar cells [83]. In line with this, Zhou and colleagues (2019) suggested that miR-30b-3p levels decrease in human subjects suffering from pneumonia and in a mouse model of LPS-induced acute lung injury [80]. Both miR-145 and miR-27a-3p can modulate lung fibrosis by targeting myofibroblast differentiation [81,84], whereas miR-302 enhances the host’s recovery from pneumonia [85]. Furthermore, mesenchymal stem cells derived EVs (MSC-EVs) can suppress ABCC1 protein expression resulting from miR-145 transfer and regulation of the leukotriene LTB4/BLT1 signaling pathway [86]. This reduces TNF-α via increased reparative macrophage M2 polarization due to miR-27a-3p transfer [87]. Epigenetic regulation in lung repair during recovery from alveolitis associated with influenza infection demonstrates that miR-155 plays a key role, as evident from the experimental results where miR-155 knockout mice recovered from influenza infection faster than control mice and also experienced decreased lung inflammation and ER stress [82]. With the urgent need for effective treatment of COVID-19 patients, a phase I clinical trial was registered in China (NCT04276987) to investigate the efficacy of mesenchymal stem cells derived exosomes (MSCs-Exo) in the treatment of severe cases of post-COVID pneumonia [88]. Altogether, miRNAs undoubtedly hold a strong therapeutic potential for epithelial cell and lung recovery from COVID-19 infection.

## 6. Epigenetic Hijacking

Epigenetics play a crucial role in host–pathogen interactions. All cells in a human organism essentially share the same genome, but they differ in expressing their genes by another wall of regulation which creates an effect on their functions and characteristics thereby leading to distinct cell lineages. Epigenetics causes changes in the phenotype which are actually the result of changes in chromosomes (sequence-specific methylation, histone, and chromosomal packing) rather than in the DNA sequence itself [89]. Epigenetic patterns are usually represented by the complex interplay between the two fundamental mechanisms of DNA methylation and histone modification, both of which coordinate together to regulate gene expression and are crucial for the normal processes of development and differentiation in higher organisms [90]. Epigenetic modifications could be of three types, DNA methylation, histone modification, and regulation by non-coding RNAs. Together, these modifications govern how the genome is regulated during development, differentiation, and during a diseased state [91,92]. In addition, they may also alter the host cell reaction to pathogens which could either delay or inhibit the immune response. This could occur innately or be caused by the pathogen itself. Notably, pathogens bring changes to the host epigenome for their own benefits thereby ensuring disease persistency and spread. These epigenetic modifications have been noticed both in the case of bacterial and viral infections [93]. In the case of certain viruses such as EBV (Epstein-Barr virus), these modifications have been reported to even induce cancer [94]. Thus, it is evident that viruses have developed various kinds of mechanisms to hijack the host epigenome, for completing their life cycles and escaping host immune responses. They mainly target epigenetic manipulations on genes that confer immune response, inflammation, survival, and cell death in order to promote replication, proliferation, and in some cases, cancer [95]. Although there is far less evidence that viruses control the ncRNA of the host to manipulate the host epigenome, the virus itself can produce different ncRNAs which have the potential to disrupt the host’s epigenome [96,97]. Apart from suppressing the host’s immune response by hijacking the epigenome, viruses can also repress epigenetic regulation to suppress their own gene expression which can lead to latent infections [98].

It has recently been demonstrated that SARS-CoV-2 also possesses the capability to manipulate the human host epigenome in order to evade the host immune response. In fact, the genome of SARS-CoV-2 potentially contains at least 50 m6A (N6-methyladenosine) modification sites. The alterations in these m6A methylation patterns of the virus can substantially affect various stages of viral infection including viral entry, replication, and host immune evasion [99]. At the same time, following a viral infection, m6A epi-transcriptome levels of the host, which normally play a role in resisting the virus, can also undergo crucial changes which affect the course of the disease progression. Some of the proteins encoded by the SARS-CoV-2 can promote such epigenetic changes in the host genome which can interfere with the innate immune signaling of the host cell [100]. Importantly, it has been reported recently that the human angiotensin-converting enzyme 2 (*ACE2*) gene, which codes for the entry receptor protein for SARS-CoV-2, also undergoes epigenetic regulation such as DNA methylation [99,101]. Another study reveals that DNA methylation patterns in the *ACE2* gene are directly associated with clinical factors for the outcome of the SARS-CoV-2 infection including age and gender [102]. Other than *ACE2* methylation, SARS-CoV-2 can also cause alteration in potent epigenetic systems such as replication behavior, antigen presentation, immune response, epigenetic signaling, and histone mimicry. However, detailed mechanisms behind these processes are yet to be investigated [103]. Furthermore, the host epigenome plays an important role in the severity of the COVID-19 infection as well. For instance, few genes involved in inflammation and immune response were found to be up regulated in severe cases of the viral infection [104]. Taken together, epigenetics such as DNA methylation patterns of SARS-CoV-2 entry receptor genes (not only *ACE2* but also coreceptors) play a pivotal role in the pathophysiology and the severity of COVID-19 disease. Thus, targeting of epigenetic modifications induced by the SARS-CoV-2 in the patient (inflammation, inflammasome, cytokines, and receptors) can help to design novel approaches for therapeutic intervention (e.g., stratification and management of patients) while direct targeting of methylation patterns is far more challenging [104]. 

## 7. How to Steer Biological Networks into the Right Direction

A disease phenotype is not just a result of the defect in a single gene, but a consequence of perturbations in the biomolecular interactions at intra- or intercellular levels. Thus, to investigate the molecular complexity in a pathological process, it is crucial to understand the biomolecular interactions governing the whole biological system rather than focusing on a single genetic mutation. In this context, network-based systems biology techniques have emerged as effective tools for understanding the complex behavior of biological systems [105]. Biological networks provide a broad multifaceted framework to simulate dynamic properties of a biological system. They employ an integrative and holistic approach to model the “interactome” so as to comprehend, characterize, and meaningfully process all the biomolecular interactions in a cell. They also depict the enrichment patterns and significant associations among several components of the biological system in order to identify novel interactions or functions by connecting the dots in the network [106]. Thus, biological networks provide a platform not only to investigate the disease modules, pathways, and mutations associated with a particular pathological state but also to uncover potential biomarkers as well as therapeutic target molecules for complex diseases. Over the last two decades, different types of biological networks such as signaling networks [107,108], gene regulatory networks [109,110], metabolic networks [111,112], host-pathogen interaction networks [113,114] and protein-protein interaction (PPI) networks [115,116] have been used for understanding drug targets [117,118] and disease mechanisms [119]. Among these, in particular, signaling networks can be efficiently controlled and steered from one state to another state by modulating the suitable targets [120,121]. Proteins dispersed in the cytoplasm are the building blocks of the cellular signaling networks, which often undergo either activation or inhibition, when a specific signal reaches into the cell. External inputs are constantly received by cellular receptors and are further processed by signaling components (such as kinases and phosphatases) followed by transcription factors to influence their cell fates.

The network-based computational approach has also been employed recently to investigate the molecular mechanisms involved in the SARS-CoV-2 infection. For instance, in a recent study, a PPI network was constructed in order to analyze the molecular basis of the effect of comorbidities such as diabetes or cardiovascular diseases on severity of progression of infection in COVID-19 patients [122]. In several different studies, information available from the drug databases has been analyzed using the network-based approaches to provide a in silico prescreening of clinically useful drugs so as to repurpose them against SARS-CoV-2 [123,124,125,126,127]. In another study, [128] a three-layered network model was prepared in order to predict protein-protein interactions between SARS-CoV-2 proteins and human proteins and identified the proteins most central to this virus–host interaction. These identified proteins were further correlated with the differentially expressed gene patterns observed in the experimental data. Messina et al. (2020) [129] also developed a network-based approach to model virus–host interactions in three previously occurring human coronavirus infections (SARS-CoV, MERS-CoV, and HCoV-229E) and used this data to predict the molecular basis of the prevalent SARS-CoV-2 infection. Based on this, they also performed structural reconstruction of SARS-CoV-2 proteins, especially the spike glycoproteins (S-glycoproteins) of SARS-CoV-2 and an interactome map was constructed to map their interactions with host cell proteins. Altogether, all these analyses highlight the significance of biological networks in gaining deeper insights into the molecular basis of host–pathogen interactions in human coronavirus infections [129]. 

**Figure 1:**
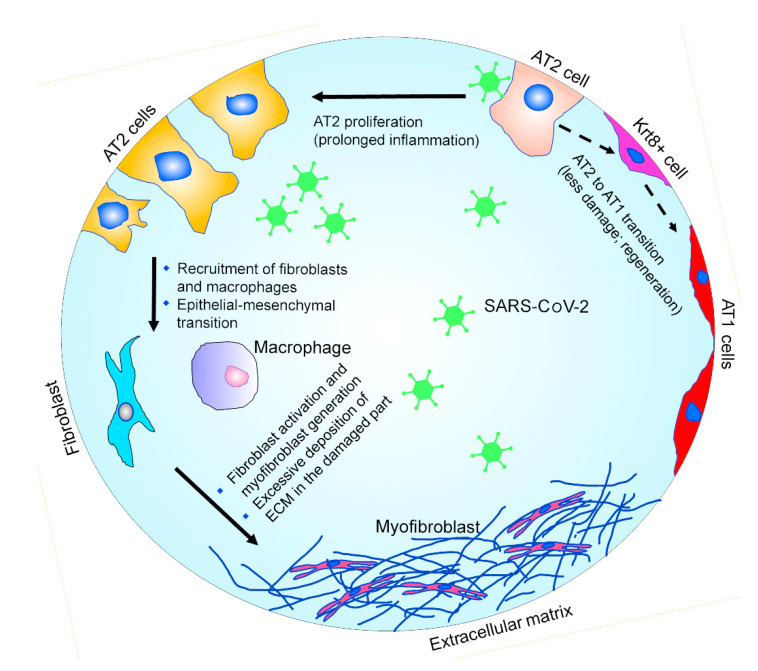
Schematic representation of the pathophysiology of alveolar repair. After injury, during the regeneration process, a subgroup of AT2 cells behave as progenitor cells and give rise to AT1 cells via an intermediate Krt8+ stage. The prolonged inflammation caused by the SARS-CoV-2 infection can trigger excessive fibrotic repair. The deposition of collagen and other extracellular matrix components during the excessive fibrotic repair plays a critical role in fibrosis.

We also highlight here how we can construct a cellular signaling network of AT2 cells to elucidate critical factors involved in the state transition from AT2 to AT1 cells. By integration of a prior knowledge network (PKN) with omics, a data condition-specific signaling network can be modelled [130,131], and by analysis of such networks, testable hypotheses can be generated [132]. The OmniPath database provides the curated global human inter- and intracellular signaling network [133]. This meta-database integrates signaling knowledge from scientific literature and more than 100 existing data resources [133] and can be used with high confidence to derive cell specific networks based on prior knowledge [134]. Using the scRNA-seq study, Strunz et al. (2020) have recently identified the Krt8+ transitional stem cells state during alveolar regeneration [17]. This state represents the intermediate state between AT2 to AT1 transition during alveolar regeneration (Figure 1). 

KeyPathwayMiner is a tool to integrate PKN with omics datasets (such as DNA microarrays, RNA-seq, scRNA-seq, etc.) to extract connected subnetworks with a high number of differentially expressed genes [135]. KeyPathwayMiner includes a set of heuristic algorithms (e.g., AI strategy ant colony optimization) and exact (fixed-parameter) methods for such integration. For a detailed explanation of KeyPathwayMiner algorithms and functionality, please refer to [130,136]. 

We hereby propose that KeyPathwayMiner [130] can be successfully used to reconstruct the condition-specific signaling network of AT2 and Krt8+ states by integrating the OmniPath signaling network [133] and scRNA-seq from Strunz et al. (2020) [17]. The resulting network can be analyzed for topological and functional characteristics [115,137,138,139]. Although there are many algorithms to analyze the network and extract desired information, we recommend using control theory [140] and in particular the ‘transittability algorithm’ proposed in [141] for analyzing the proposed AT2 and Krt8+ networks (see [140]). Classical control theory is a branch of mathematics and engineering that deals with the use of feedback control to regulate the behavior of dynamical systems. Control theory emerged from the conception and generalization of design strategies aimed at enhancing the stability, resilience, and performance of physical systems in a variety of applications [142,143]. One of the most important challenges in systems biology is the control of cellular activities by regulating specific biomolecules in a complex network. Here, control theory has evolved as a useful paradigm for studying and engineering biological systems at their optimal intervention points. According to control theory, a subset of the suitable input nodes can be sufficient to steer the signaling network from any initial state to any final state in finite time [144,145]. Control theory often studies the control of different biological processes. In this context, we focus on control theory applied to change, control and manipulate specific biological networks, in this case central pathways and the control point of the alveolar epithelium, in particular, based on the condition of an ongoing or past SARS-CoV-2 infection. 

The authors in [141] treat the problem of how to change a network from an unexpected state to a desired state and the concept of “transittability” of complex networks. They define the steering kernel as a minimal set of steering nodes to which control signals must be applied directly to transition between two specific states of a network. They introduce identification of the kernel for the desired transition between two specific states using a graph-theoretic algorithm. 

**Figure 2:**
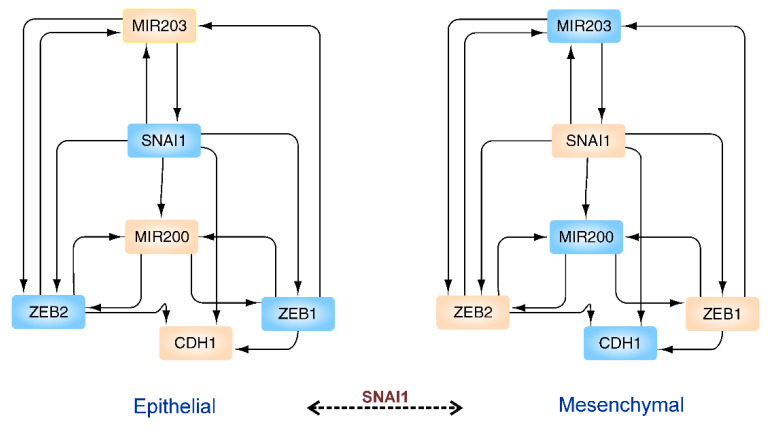
EMT network. Transition of cell phenotype from epithelial to mesenchymal state via a change in transcriptional program is shown. The color of the nodes indicates the different expression (peach = high expression; blue = low expression). The steering kernel for the transition is labelled above the dashed arrow. The network is drawn according to the data by Wu et al. [141].

In control theory, biological networks are often analyzed to identify the driver nodes, which should be actuated by control signals for acquiring control over the system under study [146,147]. On giving the end states of nodes/proteins in the network, the transittability algorithm can find out which nodes/proteins in the start network should be actuated in finite time to achieve the desired end state. For instance, in the core network regarding epithelial to mesenchymal transition, the transittability algorithm can correctly identify that SNAl1 should be actuated for EMT transition (see Figure 2) [141]. The crucial role of SNAl1 in the EMT network, as identified by the transittability algorithm, is in complete agreement with the experimental results [148].

Similarly, this algorithm can accurately identify the crucial proteins which should be modulated for phenotype transitions of p53-mediated DNA damage response network into normal, arrest, and apoptosis states (see [141] for details and more examples). As we now know the genes expressed in AT2 and Krt8+ cells [17], it is feasible to reconstruct the cellular networks of both states. Furthermore, using transittability, we can identify the proteins which should be modulated by drug molecules to trigger the AT2 state transition. Likewise, steering proteins for Krt8+ to AT1 transition can also be identified using transittability analysis. Knowing the network of AT2 and Krt8+ cells [140] will allow for the calculation of appropriate external stimuli between steady states of cellular networks [149] and, hence, will allow optimal drug application and dosage. Integrative omics data analysis [150] paves the way in the direction of lung regeneration preventing fibrosis. For example, the miR506-quaking axis and, hence, miRNAs and RNA binding protein quaking turned out to be promising targets to ameliorate lung fibrosis [150]. Control theory methods are powerful enough that you can even model the whole COVID-19 pandemic and derive estimates for exogenous drivers of the infection such as lockdowns and intervention strategies [151].

## 8. Concluding Remarks and Future Directions

The ongoing COVID-19 pandemic situation emphasizes the importance and urgency of learning more about alveolar regeneration in the face of severe acute lung injury induced by inflammation. The reasons why regeneration or fibrotic repair occurs after lung injury are evident. Delay in initiation of activated AT2 differentiation indirectly favors healing by fibrotic repair. Consequently, high level of fibrosis may ultimately lead to lung failure and death. Unfortunately, there is no fully reliable treatment strategy currently available for post COVID-19 pulmonary fibrosis. Furthermore, underlying reasons and molecular mechanisms involving pathogenesis of post-COVID lung fibrosis are poorly understood. Therefore, further research is required not only to confirm a direct connection between COVID-19 infection and pulmonary fibrosis, but also to improve mechanistic understanding of the process of COVID-19 associated fibrogenesis and factors driving it. This will also help us to identify the biomarkers that could help in the early detection of cases progressing towards pulmonary fibrosis. Moreover, it is pivotal to elucidate the effect of various risk factors such as age, gender, smoking history or comorbidities such as diabetes and hypertension on lung injury. Despite the fact that there are limited data available from direct studies in COVID-19 patients or animals, tremendous efforts have been in progress lately to establish efficient experimental models which will help in better understanding of the mechanistic details of post-COVID fibrosis. Some of these in vitro models which have been successfully employed for this purpose include lung organoids, precision-cut lung slices (PCLS), and lung-on-chip (LOC) models [52]. At the same time, it is also crucial to optimize these models for research and pharmacological intervention. Another approach that is being widely adopted is to perform an autopsy on COVID-19 patients who succumbed to the disease. We have cited in this article many recent studies on COVID-19 deceased patients, which confirm regeneration of damaged lung epithelium following infection, contrasted by other cases of extensive alveolar damage and lung fibrosis. Intriguingly, a recent study employed 420 autopsy specimens from COVID-19 deceased patients, studying exactly this infection, to construct a COVID-19 biobank which should help in the better understanding of pathophysiology of COVID-19 infection [152]. Moreover, another high-quality study investigated how SARS-CoV-2 spike protein binds ACE2 receptor only in concert with host serine proteases to promote cellular entry. Using single cell sequencing data from humans, primates, and rodents, and integrating over all datasets, applying transcriptome and pathway analysis, they showed that alveolar cell entry relies on interferon-driven stimulation of upregulation of ACE2, exploiting the fact that ACE2 works as a key tissue-protective component during acute lung injury [153]. Taken together, all these different approaches are helpful in the identification of novel strategies for prevention as well as therapeutic intervention of this disease. 

In recent years, network-based system biology approaches have been established as robust tools for studying complex biological systems. In this review, we support the view that reprogramming of progenitor AT2 cells is of central importance for triggering regeneration. Understanding and activating the AT2 transition through pharmaceutical or biological therapies can potentially accelerate the alveolar regeneration. The hijacking of the lung-protective action of ACE2 receptors boosted by interferon for virus entry is a particular challenge of the COVID-19 infection. Hence, preventing this virus entry is clearly a key handle to treat COVID-19 infection as supported by the most recent impressive and promising clinical trial results from AstraZeneca for a two-antibody entry prevention strategy (AZD7442). However, for treatment of the chronic cases (long COVID-19 syndrome) and to booster recovery from COVID-19 infection, we need and expect to uncover new regeneration-specific steering proteins and markers but, even more so, improved network control points such as specific miRNAs and epigenetic modifications as outlined here to use as improved handles that will enhance our ability to boost regeneration processes not only in COVID-19 infection but also in other chronic and acute lung diseases.

## Data Availability

All data reviewed are summarized in this publication and the references we give contain all the original data we reviewed.
